# Atomic Layer Deposition of Alumina-Coated Thin-Film Cathodes for Lithium Microbatteries

**DOI:** 10.3390/ijms241311207

**Published:** 2023-07-07

**Authors:** Aaron O’Donoghue, Micheál Shine, Ian M. Povey, James F. Rohan

**Affiliations:** Tyndall National Institute, Lee Maltings, University College Cork, T12 R5CP Cork, Ireland; aaron.odonoghue@tyndall.ie (A.O.); 114329576@umail.ucc.ie (M.S.); ian.povey@tyndall.ie (I.M.P.)

**Keywords:** thin-film microbattery, interface engineering, cathode doping, lithium metal anode

## Abstract

This work shows the electrochemical performance of sputter-deposited, binder-free lithium cobalt oxide thin films with an alumina coating deposited via atomic layer deposition for use in lithium-metal-based microbatteries. The Al_2_O_3_ coating can improve the charge–discharge kinetics and suppress the phase transition that occurs at higher potential limits where the crystalline structure of the lithium cobalt oxide is damaged due to the formation of Co^4+^, causing irreversible capacity loss. The electrochemical performance of the thin film is analysed by imposing 4.2, 4.4 and 4.5 V upper potential limits, which deliver improved performances for 3 nm of Al_2_O_3_, while also highlighting evidence of Al doping. Al_2_O_3_-coated lithium cobalt oxide of 3 nm is cycled at 147 µA cm^−2^ (~2.7 C) to an upper potential limit of 4.4 V with an initial capacity of 132 mAh g^−1^ (65.7 µAh cm^−2^ µm^−1^) and a capacity retention of 87% and 70% at cycle 100 and 400, respectively. This shows the high-rate capability and cycling benefits of a 3 nm Al_2_O_3_ coating.

## 1. Introduction

One of the challenges to improve the performance of lithium microbatteries to meet increasingly demanding energy storage requirements in a small footprint is the development of suitable electrode materials [1]. This requires improvements in both the anode and cathode with the latter being the rate-limiting electrode due to lower capacities and conductivity. Lithium cobalt oxide (LCO) has been one of the cathodes of choice since the commercialisation of Li batteries in 1991 and to date is still one of the most competitive cathode materials available due to its high theoretical capacity (140–272 mAh g^−1^), high operation potential, rate capability and life cycle [2,3].

LCO has a theoretical capacity of 272 mAh g^−1^ but a practical capacity of ~140 mAh g^−1^ where Li_1−x_CoO_2_ is limited to x = 0.5, which occurs in the potential region of 3 to 4.2 V. When >0.5 mole of Li is extracted by cycling beyond 4.2 V to 4.5 V, rapid capacity fade is observed. At these increased upper potential limits, a phase transition occurs in which Co^3+^ is oxidised to Co^4+^, resulting in an unstable phase. Co^4+^ causes damage to the crystalline structure and dissolves into the electrolyte, leading to irreversible capacity loss [4,5].

Coatings have been utilized on standard-powder-based thick-film cathodes to suppress cobalt dissolution at higher potential ranges using Al_2_O_3_ [6,7,8], MgO [9,10], AlF_3_ [11] and TiO_2_ [12]_._ These coatings are also reported to improve the charge–discharge kinetics of LCO by improving the interfacial stability, which has also been reported in other metal-oxide-based cathodes [13,14]. Alternative coating processes to ALD are also reported, such as the etching of Al_2_O_3_ on compatible substrates to obtain thin-film coatings [15]. Similarly, doping of LCO has been reported with Mn [16], Ni [17], Mg [18,19], Ti [20], La [21] and Al [7,22]. Dopants are reported to expand the c-axis when replacing cobalt, causing an improvement in lattice stability and allowing for higher-rate cycling due to easier Li^+^ diffusion and a reduction in structure breakdown at higher potentials [23,24]. 

In this work, the use of Al_2_O_3_ coatings deposited via ALD on sputter-deposited thin-film, binder-free LCO cycled to 4.2, 4.4 and 4.5 V upper potential limits is reported. A voltage of 4.2 V is investigated to show the effects Al_2_O_3_ coating has on LCO where no Co dissolution occurs. Following this, 4.5 V is investigated to compare with a previous report utilising powder and binder-based electrodes for which the ALD coating was sufficient to permit the higher potential [6]. However, this was not the case with thin-film sputter-deposited LCO. A voltage of 4.4 demonstrates the high-rate capability of Al_2_O_3_-coated LCO over 400 cycles using a practical current-rate sequence, which mimics real-world microbattery usage in medical devices, sensors and other MEMS devices. The results are consistent with Al doping, leading to improved performance by increasing stability in the crystal structure. 

## 2. Results

### 2.1. Upper Limit of 4.2 V

**Figure 1:**
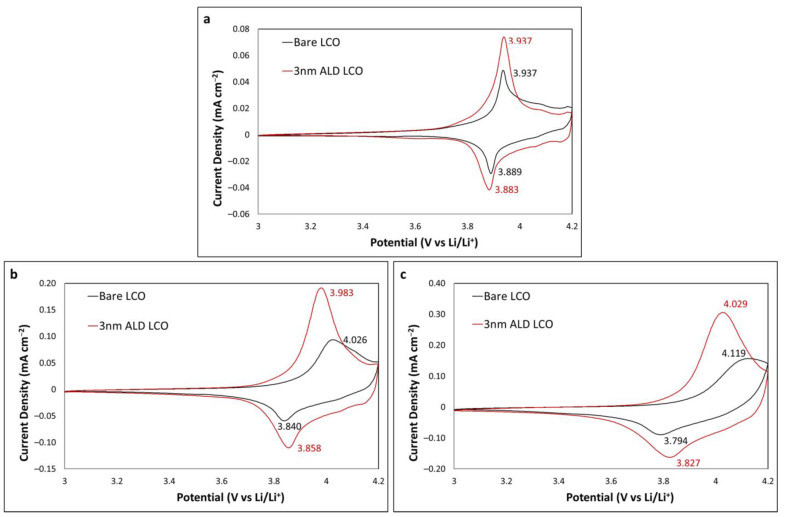
CV profiles of bare and 3 nm Al_2_O_3_-coated LCO at (**a**) 0.05 mV s^−1^ (cycle 10); (**b**) 0.2 mV s^−1^ (cycle 40); and (**c**) 0.5 mV s^−1^ (cycle 70).

CV was utilised to investigate the effect an Al_2_O_3_ coating has on LCO when the upper potential limit is 4.2 V. A 3 nm coating of Al_2_O_3_ was initially chosen as it had previously been reported in work by one of the authors in collaboration with Teranishi et al. to have the greatest improvement on the cyclability of LCO [6]. Figure 1 shows the CV profiles at scan rates of 0.05, 0.2 and 0.5 mV s^−1^ for cycles 10, 40 and 70, respectively; a large increase in peak current height is observed for the 3 nm coated LCO at all scan rates. At a slow scan rate of 0.05 mV s^−1^, the peak separations are comparable at 48 and 54 mV for bare and 3 nm coated LCO, respectively, indicating that this thickness of Al_2_O_3_, which is a resistive metal oxide, did not adversely influence the kinetics of the LCO reactions [25]. In fact, at 0.2 mV s^−1^, the peak separations are larger at the bare LCO (186 and 125 for bare and coated LCO, respectively), indicating that the ALD alumina is beneficial for reaction kinetics. The trend continues for the faster 0.5 mV s^−1^ sweep with peak separations of 325 and 202 mV for bare and 3 nm coated LCO, respectively. These results indicate that the Al_2_O_3_ coating has a positive effect on the charge–discharge kinetics of LCO at a potential range where no loss of capacity or Co dissolution is observed.

Ganesh et al. showed that a Zr dopant in LCO can affect the peak separation at varying concentrations [23]. Their XRD analysis showed that an expansion in the c-axis in the LCO hexagonal structure is observed during doping of Zr^4+^ ions, which replaces some Co^3+^ ions in vacant 3a Wyckoff sites, noting a more relaxed framework, which would allow for faster intercalation and deintercalation. This is confirmed by the reduction in peak separation or improved kinetics. Teranishi et al. suggested that because LCO was exposed to the ambient atmosphere for one month prior to ALD processing, the LCO formed reactive sites on the surface that react with TMA during ALD. This partially doped a portion of the surface with Al, accounting for the reduced peak separation at faster scan rates [6]. Alternatively, the Al_2_O_3_ being 3 nm is of similar thickness to a native SEI layer (2–5 nm), which could lead to favourable charge–discharge kinetics over bare LCO without a preformed SEI layer, although some works suggest Al_2_O_3_ ALD coatings do not act as an SEI layer at this thickness [14,26].

### 2.2. Upper Limit of 4.5 V

**Figure 2:**
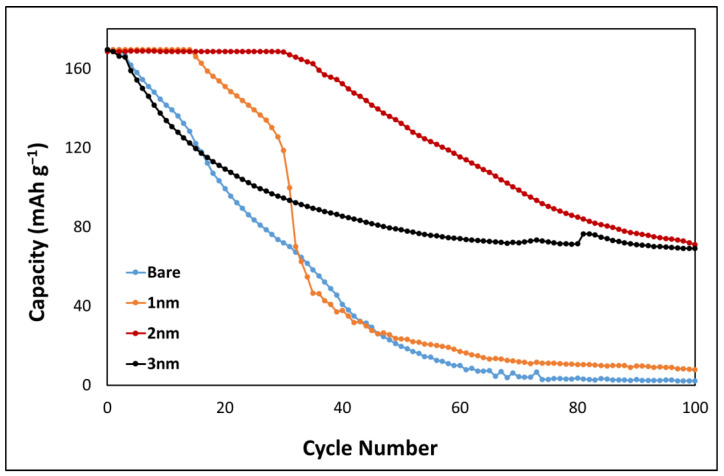
Discharge profiles of bare, 1, 2 and 3 nm coated Al_2_O_3_ at LCO at 63 μA/cm^2^.

Galvanostatic charge–discharge cycling was carried out to investigate the effect of the Al_2_O_3_ coating on the electrochemical performance of the LCO thin films at an upper potential limit of 4.5 V. A bare sample was used as a control, although it was expected to fail early due to irreversible Co dissolution. Al_2_O_3_ coatings of thicknesses 1, 2 and 3 nm were again used to compare electrochemical performance. Figure 2 shows the discharge profiles of each cell cycled at a current rate of 63 μA/cm^2^ (~1.2 C assuming a C rate of 160 mAh g^−1^). All four cells showed an initial capacity of 168 mAh g^−1^. The bare LCO showed a rapid capacity loss from the initial cycle as expected due to Co dissolution. LCO coated with 1 nm showed initial stability, but rapid capacity loss was observed at cycle 15 and showed a similar decay in capacity to the bare LCO sample. The 2 nm coated sample showed high stability at the uppermost capacity for 30 cycles before the capacity rapidly decreased to 71 mAh g^−1^ at cycle 100. The 3 nm sample behaved similar to the bare LCO sample initially but stabilised, and a capacity of 69 mAh g^−1^ was observed at cycle 100. Low final capacities at cycle 100 were observed for all samples; however, both 2 and 3 nm samples showed an improvement in electrochemical performance, indicating a suppression of Co dissolution. 

Teranishi et al. showed improvements at an upper potential limit of 4.5 V, which is not observed in the present study [6]. Their samples were fabricated from a paste consisting of LCO powder, with a PVDF binder and acetylene black conductive additive with an electrode thickness of 8.2 μm. The work reported here is for a 750 nm binder-free thin-film LCO. Lee et al. showed improved initial performance when cycling to a 4.5 V upper potential limit where they noted that the optimal thickness of Al_2_O_3_ was 1–5 nm. However, rapid capacity loss was observed from cycle 30 onward for all alumina deposits investigated [27]. Wang et al. noted that a maximum upper potential limit of 4.4 V can be used when both coatings and deliberately added dopants are employed to enhance the LCO performance [28]. Increasing the potential limit further to 4.5 V typically requires multiple modifications, such as co-doping [18,21,22] and electrolyte additives [29,30,31]. This occurs as further extraction of Li leads to structural degradation of the LCO lattice structure and some conventional organic carbon electrolytes (LiPF_6_) also decompose at 4.5 V [32,33].

### 2.3. Upper Limit of 4.4 V

**Figure 3:**
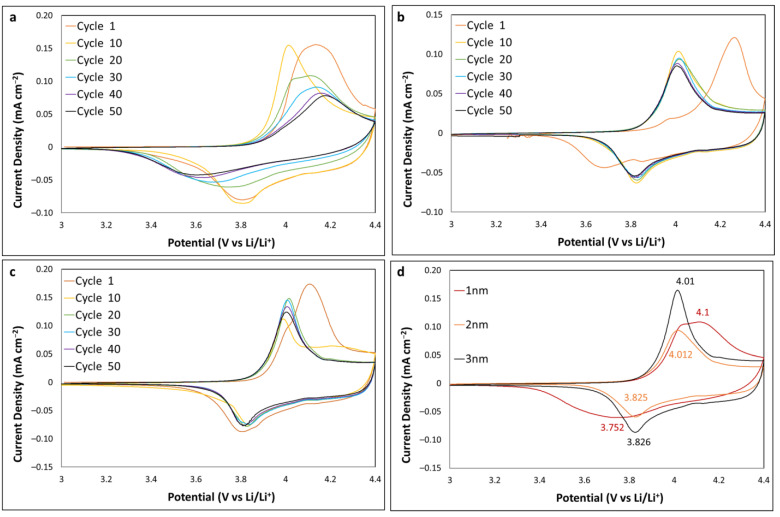
CV profiles at 0.2 mV s^−1^ for (**a**) 1 nm Al_2_O_3_-coated LCO, (**b**) 2 nm Al_2_O_3_-coated LCO, (**c**) 3 nm Al_2_O_3_-coated LCO and (**d**) overlay of cycle 20 for 1–3 nm Al_2_O_3_-coating thickness.

CV was carried out to investigate the effect of the Al_2_O_3_ coating on electrochemical performance of the LCO thin films at an upper potential limit of 4.4 V. Al_2_O_3_ coatings of 1, 2 and 3 nm thickness were used. Figure 3a–c shows the CV profiles at 0.2 mV s^−1^ over 50 cycles. The 1 nm alumina-coated LCO showed a rapid decrease in peak current following cycle 10; the broad shoulder that appeared in earlier cycles was no longer visible, and the oxidation and reduction peaks shifted to increase the peak separation from cycle 20 onwards. This is consistent with previous results in which a 1 nm Al_2_O_3_ coating was not sufficient to improve performance. The 2 nm Al_2_O_3_ film showed an improvement with the initial cycle exhibiting large peak separation, followed by identical CVs from cycle 10 to 50 with a broad shoulder being observed. The 3 nm Al_2_O_3_ film resulted in a similar performance to the 2 nm film; however, cycle 10 showed an increase in the broad shoulder portion at 4.2 to 4.3 V, with the subsequent cycles showing an increase in peak current. Figure 3d shows an overlay of cycle 20 for 1–3 nm Al_2_O_3_ coating, highlighting the peak positions with peak separations of 348, 187 and 184 mV for 1, 2 and 3 nm samples, respectively. Similar to the results for the 4.5 V upper potential limit data, both the 2 and 3 nm samples exhibit comparable results, while the 3 nm sample did show an increase in peak current height and sharpness of the peaks, illustrating improved charge–discharge kinetics.

**Figure 4:**
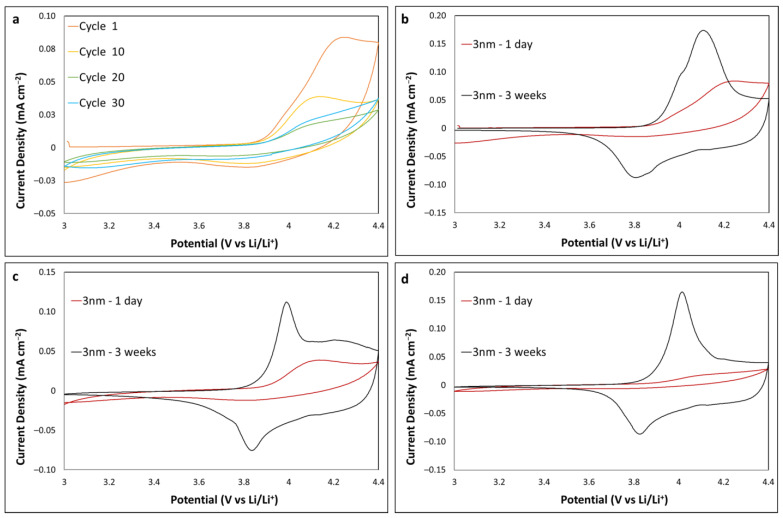
CV profiles of 3 nm Al_2_O_3_-coated LCO at 0.2 mV s^−1^ (**a**) 1-day air exposure (cycle 1 to 30), (**b**) 1-day vs. 3-week air exposure (cycle 1), (**c**) 1-day vs. 3-week air exposure (cycle 10) and (**d**) 1-day vs. 3-week air exposure (cycle 20).

Teranishi et al. noted that the LCO samples that were exposed to ambient air for a month prior to ALD may have resulted in moisture, causing reactive sites. Water is one of the reactants introduced in cycles during alumina ALD. They postulated that these sites reacted with TMA during ALD, allowing the TMA to penetrate the upper few nm and possibly further into the LCO and cause incorporation of Al into the hexagonal structure in place of Co, unintentionally doping LCO [34,35]. The LCO thin film of the present study in Figure 3 was exposed to ambient air for 3 weeks prior to both annealing and ALD (other substrates in this study were also exposed for ~3 weeks in ambient air). Figure 4 shows a comparison of CV data from Figure 3 with an LCO thin film that had 1 day of air exposure. A clear difference in CV profiles is observed. The 1-day sample showed a much larger peak separation and no broad shoulder formation, unlike the 3-week sample. The CV profiles for cycle 1, 10 and 30 are overlayed in Figure 4b–d, respectively. They highlight the poor electrochemical performance of the 1-day sample. These results further support the possibility that extended exposure to the ambient atmosphere is beneficial, assisting with Al doping the LCO and resulting in decreased peak separation and improved electrochemical performance [23]. Furthermore, this may indicate that Al_2_O_3_ coating directly on fresh thin-film sputtered LCO may adversely affect the electrochemical performance of the resistive metal oxide.

**Figure 5:**
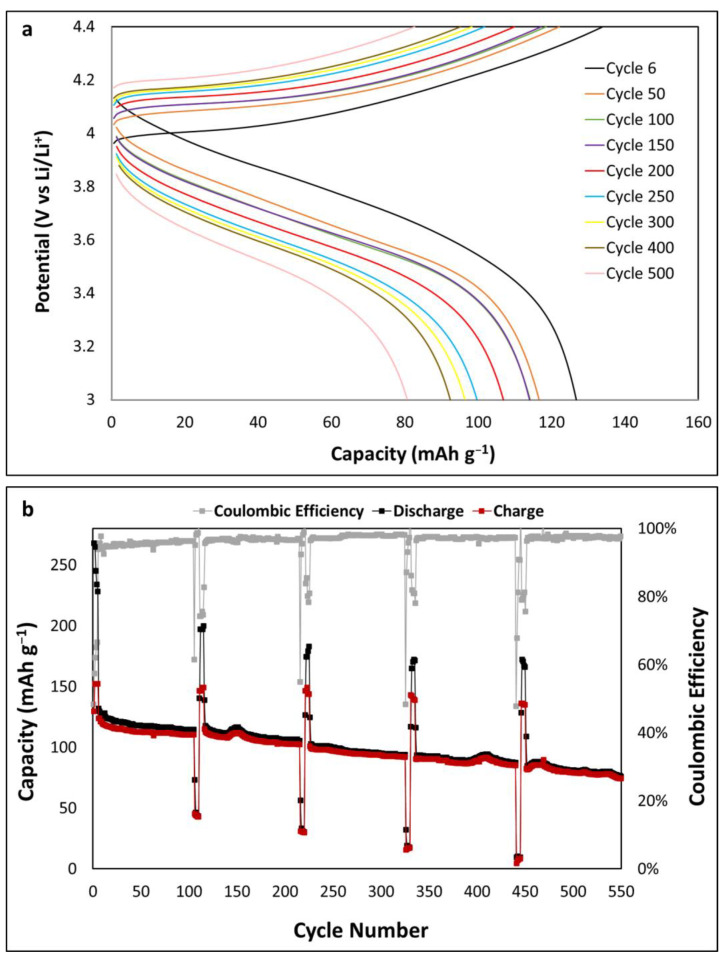
(**a**) Discharge profile of 3 nm Al_2_O_3_-coated LCO and (**b**) the measured capacity and coulombic efficiency.

Galvanostatic charge–discharge cycling was carried out to investigate long-term cycling of 3 nm Al_2_O_3_-coated LCO as shown in Figure 5. A current-rate sequence was used to simulate a device (e.g., a microsensor) where an initial low rate of 19.3 μA/cm^2^ (~0.4 C) was used for five cycles to mimic standby mode. This is followed by 100 cycles at a high current rate of 147 μA/cm^2^ (~2.7 C), which mimics data acquisition and actuation. This mode is described in more detail below. Finally, a much higher current rate of 482.5 μA/cm^2^ (~9.5 C) was used for five cycles to mimic modes, which use large current rates over a short period, such as during wireless communication of results. These modes typically last milliseconds; however, for accelerated stress testing, this mode was allowed to cycle freely for up to 12 min. Cycle 6 shown in Figure 5a is the initial discharge cycle at 2.7 C with a capacity of 132 mAh g^−1^ (65.7 µAh cm^−2^ µm^−1^). After 100 cycles, the capacity was 115 mAh g^−1^ with a capacity retention of 87%. This was followed by the larger current rate, which resulted in a large decrease in capacity as expected due to the extended time period and a large increase in the idle current rate; the capacity stabilised when it returned to 2.7 C at 115 mAh g^−1^ at cycle 120. This sequence was repeated, and the retention rates and capacities at cycle 200, 300, 400 and 500 were (81%) 107 mAh g^−1^, (72%) 95 mAh g^−1^, (70%) 92 mAh g^−1^ and (61%) 81 mAh g^−1^, respectively. These results highlight that the battery is capable of delivering intermittent high currents and achieving high-rate cycling over the tested lifetime of 550 cycles. 

The results are compared in Table 1, adapted from Bekzhanov et al., with other thin-film LCO cathodes [36]. Yoon et al. [37] and Wang et al. [38] both showed comparable results with >200 cycles but were achieved via optimization of annealing procedures, while this work illustrates an alternative method to achieve a thin film with high-rate cyclability and life cycle. Recent work from Xiao et al. showed the use of an Al co-sputter target to dope LCO. Al was sputter-deposited in an O_2_ gas flow to form an Al_2_O_3_ layer of 10 nm thickness. The thin film was cycled at 2.5 μA cm^–2^ with an initial discharge of 45.7 µAh cm^−2^ µm^−1^ for 240 cycles with 94.14% capacity retention, showing high life cycle capabilities at a very low current rate. They demonstrated high-rate capabilities with a capacity of 43.5 μAh cm^–2^ µm^–1^ at 100 μA cm^–2^, but only for five cycles from cycle 20 to 25 with no further cycling. Our work shows comparable results with alternative LCO optimisation and exceeds recent work with Al_2_O_3_ coating and Al doping. While other reported research does list adequate capacities, they do not report high cycle lifetime, which is a significant requirement for the deployment of microbatteries with thin-film cathodes designed for use in sensors, medical and MEMS devices.

## 3. Materials and Methods

LCO thin films were deposited on a stack of Au (200 nm) and Ti (10 nm) on Si coupons using a LCO sputter target (99.99% purity (Kurt J. Lesker, Hastings, UK) at a current of 150 mA and a pressure of 5 × 10^−3^ mBar in an Ar atmosphere. The deposited thin films were 750 nm in thickness, measured using a stylus surface profilometer (KLA-Tencor, Dublin, Ireland). The samples were amorphous in morphology and were subsequently annealed at 600 °C in an O_2_ atmosphere for 1 h to yield a crystalline thin film. Layers of Al_2_O_3_ that were 1, 2 and 3 nm thick were deposited via ALD following annealing using a Picosun R200 system at 150 °C. Pulse durations were 0.1 s for both trimethylaluminium (TMA) and water reagents with purge times of 4 and 6 s, respectively. Based on previous work, the alumina thin-film growth rate was assumed to be ~0.1 nm per cycle [51,52,53,54,55]. Previous studies showed that for low ALD cycles < 10 (where 1 ALD cycle ≈ 0.1 nm), an island growth phase occurs in which nucleation occurs, leading to an incomplete layer; however, >10 cycles show the formation of a continuous layer as the islands coalesce into a monolayer [56,57]. 

Electrochemical performance was assessed through cyclic voltammetry (CV) and galvanostatic charge–discharge cycling using a Biologic VSP potentiostat. A thin pouch cell was utilised with 0.25 mm thick Li (Sigma Aldrich, Schnelldorf, Germany) as a counter/reference electrode, battery grade 1M LiPF_6_ in EC:DEC (1:1) (Sigma Aldrich) was used as the electrolyte, and the surface area of the LCO cathode exposed was 0.9 cm^2^. The cells were assembled in an Ar atmosphere glovebox (MBraun LABstar, Munich, Germany).

## 4. Conclusions

In this study, the effect of ALD Al_2_O_3_ coatings on LCO cycled to 4.2, 4.4 and 4.5 V upper potential limits is shown. A 3 nm Al_2_O_3_ coating has a positive effect on the charge–discharge kinetics when compared to a bare LCO sample using an upper limit of 4.2 V. A voltage of 4.4 V not result in Co dissolution. The Al_2_O_3_ appears to partially dope LCO with Al due to reactive site reactions, assisted by exposure to ambient air prior to ALD. When LCO had limited air exposure before ALD processing, the electrochemical performance decreased and CV plots showed an increase in interfacial resistance. 

Bare LCO was compared with 1, 2 and 3 nm ALD Al_2_O_3-_coated LCO cycled to a 4.5 V upper potential limit, for which an initial capacity of 168 mAh g^−1^ were observed in both 1 nm and bare samples. They subsequently showed rapid capacity loss, while LCO with 2 and 3 nm Al_2_O_3_ showed capacities at 100 cycles of 71 and 69 mAh g^−1^, respectively. These capacities highlight a positive effect on electrochemical performance when 2 and 3 nm coatings are used.

Al_2_O_3-_coated LCO substrates of 1, 2 and 3 nm were investigated at a 4.4 V upper potential limit. CV analysis for the 3 nm coating showed the best electrochemical performance. Long-term cycling was carried out using a sequence to simulate real-world device usage with current rates for standby mode, data acquisition and communication to a readout. For the purpose of this study, the focus was on the results from the data acquisition current rate 147 μA/cm^2^ (~2.7 C). Initial discharge capacity at 2.7 C was 132 mAh g^−1^ (65.7 µAh cm^−2^ µm^−1^) with a capacity retention of 87% after 100 cycles. After cycles 200, 300, 400 and 500, the capacity retention rates were 81%, 72%, 70% and 61%, respectively, showing that this thin film is sufficient for long-term cycling beyond 500 cycles in a real-world setting.

## Figures and Tables

**Table 1:** Adapted table with data for LCO thin-film cathodes (reproduced with permission [36].

#	Material Type	Deposition Condition	Post-Treatment	Thickness	Microbattery Type	Initial Discharge Capacity	Voltage Range	Current Rate, Retention %	Cycles	Ref.
1	LCO film	Ar, DC current 150 mA	Annealed at 600 °C in O_2_ for 1 h	0.75 µm	Li/liquid electrolyte/ LCO	65.7 µAh cm^−2^ µm^−1^ (132 mAh g^−1^)	3–4.4 V	147 µA cm^−2^ (~2.7 C), 87% 147 µA cm^−2^ (~2.7 C), 70%	100 400	Our work
2	LCO film	Ar:O_2_ (3:1), heated substrate at 500 °C (in situ annealing)	-	<1 µm	Li/liquid electrolyte/ LCO	63 µAh cm^−2^ µm^−1^	3–4.2 V	1 C, 84% 1 C, 75%	100 200	[38]
3	LCO film	Ar, in situ heated substrate at 300 °C and 600 °C	Annealing by RTA 10 min at 600 °C in Ar	0.7 µm	Li/liquid electrolyte/ LCO Li/ LIPON/LCO	25 µAh cm^−2^ µm^−1^ 60 µAh cm^−2^ µm^−1^	3–4.2 V 3–4.2 V	1 C, 85% 5 C, 100%	50 100	[37]
4	LCO film	Ar:O_2_ (3:1) and (5:1), DC power 130 W	Annealed at 500 °C in atmosphere	-	Li/liquid electrolyte/ LCO	46 µAh cm^−2^ µm^−1^	3–4.2 V	0.1 C, 8.2%	100	[39]
5	LCO film	Ar:O_2_ (96:4%),	Annealed at 800 °C in air	10 µm	Li/LIPON/ LCO	60 µAh cm^−2^ µm^−1^	3–4.2 V	0.1 C, 95%	100	[40]
6	LCO film	Ar	Annealed at 550 °C, holding time 20 min at O_2_	1.1 µm	Li/liquid electrolyte/ LCO	37.5 µAh cm^−2^ µm^−1^	3–4.2 V	0.1 C, 3.8%	50	[41]
7	LCO film	Ar:O_2_ (1:2, 1:1, and 2:1), RF power 120, 150, and 180 W	1 h at 700 °C in air	1.6 µm	Li/liquid electrolyte/ LCO	16.7 µAh cm^−2^ µm^−1^	3–4.2 V	0.2 C	20	[42]
8	LCO film	Ar, laser-patterned	400 °C and 600 °C in Ar:O_2_ (1:5) 3 h	3 µm	Li/liquid electrolyte/ LCO	140 mAh g^−1^	3–4.2 V	0.05 C, 67%	30	[43]
9	LCO film	Ar:O_2_, in situ substrate heated at 250 °C	In O_2_ two hours 500 °C 600 °C 700 °C	>1 µm	Li/liquid electrolyte/ LCO	41.8 µAh cm^−2^ µm^−1^ 52.6 µAh cm^−2^ µm^−1^ 61.2 µAh cm^−2^ µm^−1^	3–4.25 V	10 µA cm^−2^, 58%, 72% 74%	50	[44]
10	LCO film	Ar:O_2_ (3:1), different deposition pressure parameters changed	500 °C 2 h in air	<1 µm	Li/liquid electrolyte/ LCO	67 µAh cm^−2^ µm^−1^	3–4.2 V	0.2 C, 95%	50	[45]
11	Zr-doped LCO film	Ar:O_2_ (9:1), in situ substrate heated at 250 °C	600 °C 3 h in air	>1 µm	Li/liquid electrolyte/ LCO	64 µAh cm^−2^ µm^−1^	3–4.2 V	1 C, 98.5%	25	[23]
12	LCO film	Ar,	400–700 °C in O_2_	<1 µm	Li/LIPON/ LCO	40 µAh cm^−2^ µm^−1^ (80 mAh g^−1^)	3.3–4.2 V	0.01 C, 78%	5	[46]
13	LCO film	Ar:O_2_ (4:1), DC power 180 W	600 °C in O_2_	0.5 µm		30.7 µAh cm^−2^ (or 56.9 µAh cm^−2^ µm^−1^)	3–4.2 V	10 µA cm^−2^, 76%	30	[47]
14	LCO film	Ar:O_2_ (3:1), RF power 100 W, in situ-heated substrate 400 °C	-	0.4 µm	Li/liquid electrolyte/ LCO	54.5 µAh cm^−2^ µm^−1^	3–4.2 V	10 µA cm^−2^, 58.20%	50	[48]
15	ZrO_2_ coated LCO film	Ar:O_2_ (4:1), DC power 100 W	600 °C 1 h in O_2_	0.6 µm	Li/liquid electrolyte/ LCO	12.2 µAh cm^−2^ µm^−1^	3–4.5 V	10 µA cm^−2^, 75%	40	[49]
16	LCO film	Ar:O2	300–700 °C 1 h in air	>1 µm	Li/liquid electrolyte/ LCO	132 mAh g^−1^ (or 62 µAh cm^−2^ µm^−1^)	3–4.3 V	0.1 C, 70%	50	[50]
17	LCO film	Ar:O_2_ (5:1), RF power 100 W	550 °C, 1 h 20 min annealed in argon	1.2 µm	Li/liquid electrolyte/ LCO	135 mAh g^−1^ (50 µAh cm^−2^ µm^−1^) 135 mAh g^−1^ (50 µAh cm^−2^ µm^−1^) 115 mAh g^−1^ (42 µAh cm^−2^ µm^−1^)	3–4.2 V	0.1 C, 93% 0.5 C, 77% 1 C, 50%	20 100 100	[36]
18	Al_2_O_3-_coated and Al-doped LCO film	Ar, RF-DC, (Al doping = 10 W DC), (Al_2_O_3_ coating = 80 W DC with O_2_ gas step) (LCO = 200 W RF) in situ substrate heated at 800 °C	-	0.5 µm	Li/liquid electrolyte/ LCO	45.7 µAh cm^−2^ µm^−1^	3–4.2 V	2.5 μA cm^–2,^ 94.14%	240	[7]

## Data Availability

The data presented in this study are available on request from the corresponding author.
